# Assessment of the Composition of Forest Waste in Terms of Its Further Use

**DOI:** 10.3390/ma14040973

**Published:** 2021-02-18

**Authors:** Marta Bożym, Arkadiusz Gendek, Grzegorz Siemiątkowski, Monika Aniszewska, Jan Malaťák

**Affiliations:** 1 Department of Process and Environmental Engineering, Faculty of Mechanical Engineering, Opole University of Technology, Prószkowska 76 Street, 45-758 Opole, Poland; 2 Department of Biosystems Engineering, Institute of Mechanical Engineering, Warsaw University of Life Sciences—SGGW, Nowoursynowska 164, 02-787 Warsaw, Poland; arkadiusz_gendek@sggw.edu.pl (A.G.); monika_aniszewska@sggw.edu.pl (M.A.); 3 Łukasiewicz Research Network—Institute of Ceramics and Building Materials, Cementowa 8 Street, 31-983 Cracow, Poland; g.siemiatkowski@icimb.pl; 4 Faculty of Engineering, Czech University of Life Sciences Prague, Kamycka 129, 165 21 Prague 6, Czech Republic; malatak@tf.czu.cz

**Keywords:** forest residues, cones, wood, calorific value, heavy metals, alkali metals, adsorbents

## Abstract

This paper presents the results of the analysis of the chemical composition and content of heavy metal contamination in forest logging residues, in order to assess the possibility for their further utilisation. The samples were divided into 9 groups, which included coniferous tree cones, wood, and other multi-species logging residues. The elementary composition, ash content, and calorific value were determined as energy use indicators for the samples. Additionally, the content of heavy and alkali metals, which may affect combustion processes and pollutant emissions, was tested. The high content of heavy metals may also disqualify these residues for other uses. The research shows that the test residues are suitable for energy use due to their high calorific value and low content of heavy metals. However, an increased ash content in some samples and the presence of alkali metals, causing high-temperature corrosion of boilers, may disqualify them as a potential fuel in the combustion process. The forest residues may be used in other thermal processes such as pyrolysis or gasification. A low content of heavy metals and a high content of organic matter permit the use of these residues for the production of adsorbents or composite materials.

## 1. Introduction

Logging residues, also known as forestry residues, have a heterogeneous composition due to the different types of material that constitute their mass. They fall into different categories such as round wood pieces, branches, bark, cones, and leaves/needles. Residues may be shredded in a loose state into chips. The utilisation of these residues may pose difficulties, but the literature describes many possibilities for their use.

In 2018, biomass accounted for 15.2% of total household energy consumption in the European Union (EU). Taking into account the Polish resources of 4 million tonnes of waste wood, it can be concluded that forest residue is misspent through inefficient use and unsustainable exploitation [1].

In Europe, the dominant application of forest waste is combustion, co-incineration, or fuel conversion (gasification, pyrolysis, torrefaction). The use in biogas plants is currently at the research stage, as waste requires pre-treatment and this reduces its attractiveness compared to agri-food waste [2]. In addition to energy and adsorbents production, forest waste may be used for the production of other biomaterials, such as wood–plastic composites (WPC) and wood-based composites, as well as the production of cellulose nanoparticles (CN), including cellulose nanocrystals (CNC) and cellulose nanofibers (CNF) [3].

Forest biomass residues are a suitable option for the development of biomaterials and biofuels in the current age of climate change and global warming. The disadvantage of using forest waste is its heterogeneity, which reduces the quality of the products. However, their availability and low price ensure the wide use of forest waste. Valorisation of forest waste is definitely more beneficial compared to wood, for both the economy and the environment [3]. 

The total global supply of biomass from agriculture and forestry is estimated at about 11.9 billion tonnes of dry matter per year, 39% of which is produced through forestry. From the total world production of wood biomass, firewood (including for energy production) accounts for 23%, while primary biomass (industrial roundwood) accounts for 8%, and losses for 8% [4].

From the point of view of the chemical industry based on biomass, the development of second-generation biofuels is a new direction. However, second-generation biofuels are currently under development, with several pilot plants in the European Union. It is possible that these technologies may become a predominant element of the economic potential of forest residues in the future [2].

Bark is a cheap and widely available by-product from both forestry and the pulp and wood industries. It may be used directly as a fuel source, soil, or mulching additive [5], and, to some extent, in the production of furniture particleboards [6,7]. It also has great potential as an effective and inexpensive sorbent for removing pollutants from water or wastewater or for gas purification [8,9,10]. The bark of some species of the conifers found in forests, due to their porous structure similar to lichens and mosses, may be used for air pollution monitoring, especially for heavy metals [11,12,13]. In order to improve bark sorption properties, mineral additives or microorganisms, so-called biosorbents, are used [14]. In Europe, the resources of coniferous (spruce and pine) bark residues are estimated at approximately 36.2 million m^3^, and 14.9 million m^3^ of deciduous trees, including conifers, in Poland about 526 thousand m^3^, in the Czech Republic about 1.1 million m^3^, in Germany about 4.5 million m^3^, and in other Scandinavian countries (Sweden, Finland), approximately 12.1 million m^3^ [2].

Other commonly used forestry residues include coniferous tree cones, notably pine and spruce. They are used for decorative, medicinal, cosmetic, and aromatherapeutic purposes. The literature also describes the use of their extracts as plant protection products [15]. As with bark, cones are also used as natural sorbents. However, they require chemical pre-treatment to improve their sorption properties by activating appropriate functional groups and removing phenolic substances, polysaccharides, and tannins that impede adsorption and colour the water [16]. Bark- or cone-based active carbons were also found to have favourable sorption properties, in particular with respect to organic compound adsorption [17]. Sample applications of various forestry residues used as sorbents of aqueous solutions pollutions are shown in Table 1.

Due to their high lignocellulose [18] and tannins content, bark and cones are used in the chemical industry and for the production of new materials such as biopolymers or composite materials [2]. In polymeric composites, additional bark or cones decrease density, improve strength and stiffness, strengthen insulation properties, reduce production costs, and increase their biodegradability [19,20]. García-García et al. [21] suggested an interesting use for pinecones. They described the production and use of cellulose nanocrystals (CNC) as materials used to strengthen composites or for food packaging. Sirén [22] described another application, suggesting a method for extracting organic acids from spruce and pine bark for further use in the food, fuel, or pharmaceutical and medical industries; also, Gendek et al. [23] proposed to crush the cones after peeling the seeds to make briquettes from them. 

**Table 1:** Examples of the use of forest waste as pollutant adsorbents.

No.	Material	Removed Substance	References
1.	Raw pinecones	Methylene Blue	[24]
		Acid Black 26 (AB26), Acid Green 25 (AG25), Acid Blue 7 (AB7)	[25]
		Cd, Pb	[16]
		Phenol and chlorophenols (CPh)	[26]
2.	Modified pinecones	Pb, Ni, Co, As	[27,28,29]
		Phosphates and nitrates	[30]
3.	Raw and activated pinecone powder	Methylene Blue	[31]
4.	Composites of cones and mineral materials	Cr(VI)	[32,33]
5.	A coagulant based on pinecones	Turbid water	[34]
6.	Modified pine bark	Reactive dye	[35]
		Cu, Ni, Zn, Pb	[36]
		As	[37]
		N, Cr	[38,39]
		Cu, Ni, Zn	[40]
		Organochlorine pesticides	[41]
		Polycyclic aromatic hydrocarbons	[42]
		Bisphenol	[43]
7.	Modified spruce bark	Fe, Pb, Cu, Cd, Zn, Ni, Ba, Ca, Sr, Mn, Mg, K, Na, Li	[6]
8.	Pine bark compost	Methylene blue	[44]
9.	Sawdust and pine bark, spruce bark	Nitrates	[45]
10.	Biosorbent based on pine bark and microalgae	Cu, Pb, Zn, Cd, Co, Ni	[46]
11.	Biochar from pinecones	As(III)	[7]
12.	Waste wood-activated carbon	Polycyclic aromatic hydrocarbons	[47]
13.	Pinecones biochar	Pb	[48]
14.	Pine bark biochar	Cu, Cr and Zn	[49]
15.	Activated carbon from pinecone	Congo Red Dye	[50]
		Acid Blue 113, Acid Black 1	[51]
16.	Pinecone-activated carbon with ZnCl2	Methylene Blue	[52]

Forestry residues are also used for energy purposes, both in a treated and untreated form, for refined fuels [53,54], and after thermal pre-treatment. The utilisation of forest biomass residues for bioenergy production is a valuable alternative to fossil fuel combustion [3]. Forestry residues may be used to generate energy through direct co-combustion with conventional fuel or indirect by gasification and afterburning or by parallel co-combustion, for instance, in hybrid systems [55,56].

Due to its high content of Ca and Mg, forestry residue combustion or co-combustion ash may be further used for the production of building materials [57], sewage or sludge neutralisation, or for use as a fertiliser [58]. The purpose of the thermal conversion of forestry residues is to produce biocarbon, bio-oil, or synthesis gas [3]. Among the most popular methods of conversion of this biomass are torrefaction, gasification, and pyrolysis [1,59,60,61]. Sobek and Werle [1] put forward an interesting solution involving the use of solar energy to reduce pyrolysis costs.

The aim of this study was to assess alkali metals’ content and the degree of heavy metal contamination of forestry residues as indicators for the directions of their further use, according to their type and species composition.

## 2. Materials and Methods 

### 2.1. Material and Sampling

Cones of conifers (samples No. 1–3) were obtained after the seed husk process from the plant in Czarna Białostocka, Poland. Chips made of lump wood, round wood, and logging residues (samples No. 4–9) were obtained as part of cleaning up forest areas located in north-eastern Poland. For sample No. 7, before chipping, the residues were additionally baling on the forest area. The obtained material was fragmented with a Bruks 805 CT (Bruks Siwertell Group, Stockholm, Sweden) self-propelled chipper. A detailed description of the samples, designation, processing site, and species composition is summarised in Table 2.

For each sample, 100 L of material were taken—10 L at random from 10 different places of the pile and mixed. Before the tests, approximately 3 L of sample were taken from each batch of material. The material was dried in a Heraeus UT6120 laboratory dryer (Kendro Laboratory Products GmbH, Hanau, Germany) at a temperature of 105 ± 2 °C and then ground in a SM100 mill (Retsch GmbH, Haan, Germany) to obtain particles <1.0 mm.

### 2.2. Methods

Elemental composition (C, H, N, S) was analysed using “CHNS Analyzer” (Elementar Analysesysteme Gmbh, Hanau, Germany) PN-G-04571 [62] and PN-G-04584 [63]. The ash content was obtained by incineration at 550 °C in a muffle furnace (Nabertherm GmbH, Lilienthal/Bremen, Germany) until constant weight [64,65]. The total content of heavy metals (Cd, Pb, Cu, Zn, Cr, Ni, Mo, Co, Fe, Mn) and nutrients (Mg, Ca, K, Na) were prepared and analysed according to Standards ISO 16967 [66] and ISO 16968 [67]. The digestion was carried out using a microwave oven (Ethos Start D, Milestone, Sorisole, Italy) in Teflon vessels with aqua regia (36% HCl Tracepur^®^ and 65% HNO_3_ Suprapur^®^, Merck, Darmstadt, Germany). Heavy metals and nutrients were determined using the FAAS method with spectrometer Solaar 6 (Thermo Electron Corporation, Cambridge, UK). Mercury content was determined using the CVAAS method using an AMA 254 analyser (Altec Ltd., Praga, Czech Republic). The calorific value was determined with the use of the KL–12Mn calorimeter (Precyzja–Bit PPHU Ltd., Bydgoszcz, Poland) [68].

### 2.3. Quality Control

Quality control was carried out using certified materials: ‘Lichen (trace elements)’ (IRMM, No. BCR–482) and ‘AlfaAlfa B2273’ (Cert. No. 41505). The control of the analytical range was based on the analysis of the certified solution ‘ICP–multielement standard XI’ (Lot: HC394644, Merck, Darmstadt, Germany). 

### 2.4. Statistical Analysis

All analyses were performed in a minimum of three replications. Mean values and standard deviations (SD) were calculated using the Statistica program (ver. 13.3, TIBCO Software Inc.). One-way analysis of variance (ANOVA) was performed to determine the differences between the mean values. Tukey’s HSD (Honest Significant Difference) multiple comparison test (post-hoc test) was used to determine homogeneous groups. All analyses were performed at the significance level α = 0.05. Homogeneous groups of means are marked in Table 3 and Table 4 with letters a, b, c.

## 3. Results and Discussion

Table 3 shows the results of the elemental composition (C, H, N, S), ash content (Ash), and calorific value (Q_i_) of the tested samples, while Table 4 shows the content of heavy and alkali metals in the samples. Due to the high content of organic matter in forest waste, carbon occurs mainly in its organic carbon form. According to a number of authors, carbon content varies by between 45–55% [5,45,50,60,69,70,71,72]. The carbon (C) content depends on the plant species and parts sampled. For instance, Ward et al. [59] found a carbon content of 69.2% in pine chips, and Kumar et al. [26] found a C content of 76.58% in pinecones. On the other hand, Frank and Cox [73] determined the carbon content in the wood of fir species (*Abies concolor*, *Abies lasiocarpa*) to be in the range of 60–63%, and the result was slightly lower in pine wood. In the tested cones (No. 1–3), carbon content ranged from 37.46–46.52%. In bark-free piece wood (No. 4) and round wood with bark S4 (No. 5), this content was slightly higher and amounted to 50.12 and 49.48%, respectively. While in other forestry residues (No. 6–9), C content ranged from 41.10–47.37%. The percentage of carbon in forest waste can vary and depends on the type of material. Carbon is the main component in organic compounds such as cellulose, hemicellulose, and lignin, but also tannins, resins, pectins, and phenolic acids. The last two compounds are important in the adsorption process, whereas cellulose and lignin compounds should be pre-treated before use as adsorbents [6]. 

The high carbon content of the waste may also increase its calorific value. Carbon compounds can be converted into fuel by pyrolysis or be burned directly. However, the loss on ignition (LOI) and the calorific value are more useful for assessing the properties of waste as fuels. In all of the tested samples, hydrogen content ranged between 5.37–6.62%. Other authors estimated that the content of hydrogen is 5–9% in wood residues [45,48,50,56,60,69,70,71,72,73]. For the tested samples, nitrogen content ranged from 0.6–0.8% in cones (No. 1–3), from 0.8–0.85% in wood (No. 4–5), and from 0.9–1.3% in other forestry residues (No. 6–9). According to literature data, nitrogen content may vary from 0.1% [71,72] to as much as 6.5% [26] in forestry residues. A high cone nitrogen content may be a consequence of the presence of protein-rich seeds [74]. Nitrogen is a component of amino acids and proteins. Usually, these compounds are removed during the processing of forest waste by adding hydroxides and organic extractants. However, the nitrogen compounds present in the waste should not have an impact on the sorption properties. Sulphur content in forestry residue may be of natural or pollution origin, e.g., SO_2_ may be absorbed from the air by the plant or it may be present in dust that was deposited on coniferous tree cones or bark [50,75,76,77]. There is usually a low content of this element (0.01–0.05%) in forest waste [69,70,71,72]. However, Khalili et al. [72] and Dawood et al. [50] reported higher concentrations of this element in forestry residues (1.2–3%). In the test samples, sulphur content was low (<0.005–0.12%), which will not affect the quality of this waste. The presence of sulphur in forest waste may indicate the adsorption of SO_2_ from the air by the bark, which is a component of the waste. However, this should not affect the adsorption properties of the waste. High sulphur content in forest waste may have a negative effect on the high-temperature corrosion of boilers during combustion and, consequently, increase SO_2_ emissions.

The content of organic compounds in wood waste is described by the loss on ignition (LOI). A high LOI value indicates a high content of organic compounds that may be used as adsorbents. On the other hand, the LOI also indicates the ash content, i.e., the inorganic compounds, which is important for waste combustion. The post-burning residue consists of ash, the content of which may vary in forestry residues. Typically, ash contains significant amounts of calcium, magnesium, sodium, and potassium, the so-called alkali elements, which may affect the rate of high-temperature boiler corrosion during biomass combustion [78,79,80]. Furthermore, the amount of ash is important for the energy use of forest residues considering the need for its management. A high content of alkali metals and a high ash pH may facilitate its use for fertiliser purposes or soil or wastewater neutralisation purposes [58] and also its use in construction [57]. The ash content varied between the tested samples. The lowest content of ash was found in cones (No. 1–3) and wood samples (No. 4–5) (1–2%). By contrast, a higher ash content (2–5%) was found in the multi-species forestry residues (No. 6–9). The ash content in forest material may vary widely. Other authors have determined a wide range of ash content in pinecone: 0.7% [54,81], 2% [28,33], 4.89% [26], and 15% [48]. According to Malaťák et al. [54], the spruce and larch cone ash content was 1.3% and 1.2%, respectively. Usually, coniferous tree bark ash content is found to be 10% or less [56,60]. According to Gendek et al. [82], the multi-species logging residues chips ash content is between 1.4–5.8%, and the authors link these results to species composition and harvesting technology. Ward et al. [59] found a high ash content (13.8%) in pine woodchips.

The calorific values of the samples were similar and ranged from 18.5–19.5 MJ/kg. A lower pinecone calorific value was found by other authors, i.e., 18.6 [81] and 17.6 MJ/kg [83]; while according to Aniszewska et al. [74], the calorific value of conifer seeds was 20 MJ/kg. Normally, the pine wood calorific value varies between 19.2 and 21.2 MJ/kg [74]. In contrast, Gendek et al. [82] found that the calorific value was dependent on the ash content of the wood residue wood chips, with results ranging from 16.3 to 18.8 MJ/kg. The heat of combustion, humidity, hydrogen content, and ash content influence the calorific value. 

Heavy metals, the content of which is usually normalised in the environment, include Cd, Pb, Cu, Zn, Cr, Ni, and Hg. Some of them are micronutrients, essential for plant life, with the exception of Cd, Pb, and Hg [13,84]. In wood residues, heavy metals may be derived from natural assimilation by roots or from dust that deposits on the outer tree parts such as bark, needles, and cones [11,12,13,74]. The content of heavy metals depends on the age and anatomical part of the plant and additionally on environmental pollution, mainly dust concentration [13]. Therefore, an increased content of atmospheric dust-derived metals is found in coniferous tree needles, cones, or bark [75,85].

Mercury is considered to be one of the most toxic heavy metals [84]. Plants absorb mercury from the soil through the root system, or directly from the air. In the tested samples, the content of this metal was very low, and varied between 0.005–0.010 mg/kg. Similarly, Aniszewska et al. [74] found a very low level of pine and spruce seeds Hg content (0.002–0.003 mg/kg), which may be the result of a limited penetration of heavy metals into the seed material [84]. By contrast, Aboal et al. [86] found a higher level of pine needles mercury with a content of 0.006–0.032 mg/kg, and Galhetas et al. [55] found a pine wood residue mercury content of 0.021 mg/kg. 

In most of the tested samples, cadmium content was below the detection limit (<0.5 mg/kg), with the exception of sample No. 9.

It may therefore be concluded that the test samples were not contaminated with this metal. For comparison, Aniszewska et al. [74] found a high Cd content of 1.6 mg/kg in pine and spruce seeds. The authors also found a higher content of this metal in seed wings, which was attributed by them to the large surface area, which affected the accumulation of contaminated dust. Kirchner et al. [87] found a pine wood cadmium content of 0.02–0.17 mg/kg, and Poikolainen [88] found a cadmium content of 0.10–0.23 mg/kg in the bark of pine trees growing in areas with different degrees of contamination.

The lead content in the tested material varied. In cones (No. 1–3), a lead content of 1.5–10.5 mg/kg was found. In bark-free wood (No. 4), a low content of Pb (2.4 mg/kg) was found. The sample of round pine wood with bark (No. 5) was characterised by a very high content of lead (150 mg/kg). Dust deposited on the bark may have been the probable source of sample contamination. In other residues (No. 6–9), a lead content of 74.6–153 mg/kg was found. The increased Pb content in some tested samples may have been caused by environmental pollution with this metal. Other authors found a lower lead content level of 2.80–8.45 mg/kg in spruce branches [89], 15.23–54.26 mg/kg in pine needles [77], and 0.98–3.94 mg/kg in pine bark [88], respectively. 

Copper is a component of many plant enzymes [90]. Conifers are resistant to high Cu environmental concentrations and may accumulate significant amounts of this metal [87]. In the test cones (No. 1–3), a copper content of 2.3–3.2 mg/kg was found. The lowest Cu content (1.7 mg/kg) was found in bark-free pine wood (No. 4). A higher content of copper was found in round wood with bark (No. 5). In other residues (No. 6–9), the copper level varied between 4.4 and 7.1 mg/kg. Other authors noted a Cu content in pine bark of 2.5 mg/kg [10] and of 2.54–4.03 mg/kg [88]. Aniszewska et al. [74] found a pine and spruce seeds Cu content of 9.93 mg/kg. For comparison, Nkongolo et al. [89] found a Cu content in spruce branches in the range of 11–28 mg/kg, and Sawidis et al. [77] determined a content of 52–123 mg/kg in pine needles. 

In the environment, the proportion of zinc is usually high compared to other heavy metals [84]. Like copper, zinc belongs to the group of microelements. Zinc is a component of enzymes and plant growth regulators, and is present to a significant extent in auxins and indoleacetic acid (IAA) [90]. In the tested cones (No. 1–3), zinc content varied between 12.2 and 22.6 mg/kg. A lower zinc content (10.8–11.7 mg/kg) was found in pine wood (No. 4–5). In other residues (No. 6–9), Zn content varied over a wide range from 11.7 to 58.1 mg/kg. Other authors also found that zinc varied within a wide range in different parts of pine and spruce trees, i.e., 30.33–52.33 [89], 11.89–24.67 [87], and 14.7–20.3 mg/kg [88]. A higher Zn content of 83.4 mg/kg was found by Aniszewska et al. [74] in the seeds of pine and spruce. 

Nickel is a plant microelement present in plant enzymes, mainly urease and hydrogenases [84]. Coniferous trees are resistant to increased environmental levels of nickel, and pine trees belong to the class of Ni hyperaccumulators [87]. In the tested cones (No. 1–3), nickel content varied from 1.4 to 3.5 mg/kg. In bark-free pine wood (No. 4), Ni content was below the detection limit. The highest content of nickel (9.3 mg/kg) was found in round wood with bark (No. 5). In other samples (No. 6–9), the content of this metal varied between 3.4–6.4 mg/kg. Poikolainen [88] found a Ni content in pine bark of 0.79–2.43 mg/kg. 

Other authors noted a higher Ni level: 7.56–36.37 mg/kg in pine branches [89], 9.37–24.30 mg/kg in pine needles [77], and 13.16 mg/kg in pine and spruce seeds [74], respectively.

Chromium also belongs to the group of microelements [84]. Chromium content in tested cones (No. 1–3) was approximately 3 mg/kg. A similar content of Cr was found in pine wood (No. 4) (3.2 mg/kg). The highest content of Cr was found in sample No. 5 (31.4 mg/kg), i.e., round wood with bark. In other residues (No. 6–9), the chromium content varied from 11.2–16.3 mg/kg. Kirchner et al. [87] determined a very low Cr level (<1 mg/kg) in pine wood (*Pinus jeffreyi*). Similarly, Liu et al. [10] and Poikolainen [88] found a Cr content in pine bark of 0.5 and 0.12–7.31 mg/kg, respectively. Aniszewska et al. [74] noted a chromium content of 8.27 mg/kg in pine and spruce seeds. Sawidis et al. [77] found a chromium content of 8.33–23.53 mg/kg in pine needles. 

To compare the content of heavy metals in the tested forest wastes used for energy and fertilisation purposes, Table 5 is presented, which presents the limits for composts and biofuels according to Polish standards. Usually, forest waste is not used directly as fertilisers, only after treatment, i.e., composting process. Due to the high content of cellulose, forest waste is used as structure-forming material in the composting process. The standard [91] is used to evaluate quality of compost from municipal waste and other types of waste, e.g., sewage sludge in Poland [92]. In wood waste, including logging residues, used for energy purposes as wood chips, the content of heavy metals is not standardised. However, in chips from other wood waste (class B1 and B2) as well as pellets and briquettes (class A1), the content of heavy metals is standardised (Table 5).

The tested waste did not exceed the permitted heavy metal limits specified for the first class of compost. On the other hand, other forestry residues (No. 6–9) exceeded the limit for chromium content for solid biofuels. This effect could be caused by a secondary mineral contamination of the waste during processing or storage. Other metals (Mo, Co, Fe, Mn) are not monitored in forest waste used as fertiliser or for energy purposes in Poland.

Molybdenum is a component of enzymes involved in nitrogen metabolism in plants [83]. In cone samples (No. 1–3), molybdenum content ranged from 11 to 19 mg/kg. In contrast to other metals, molybdenum content in wood (No. 4) was higher (29 mg/kg) than in cones. 

In other samples (No. 5–9), molybdenum content ranged from 15 to 32 mg/kg. Kirchner et al. [87] found an Mo content of <1 mg/kg in pine wood (*Pinus jeffreyi*). Aniszewska et al. [74] noted a molybdenum content of 33 mg/kg in pine and spruce seeds. 

Cobalt is a micronutrient, which influences the processes of atmospheric nitrogen fixation in plants [84]. In tested cone samples (No. 1–3) and round wood with bark (No. 5), cobalt content was found to be below the detection limit. In other samples, the cobalt content varied between 1.0 and 1.9 mg/kg. For comparison, Kirchner et al. [87] found a Co content <1 mg/kg in pine wood (*Pinus jeffreyi*). Likewise, Poikolainen [88] found a low Co content (0.06–0.11 mg/kg) in pine bark. Aniszewska et al. [74] noted a Co content of 1.34 mg/kg in pine and spruce seeds.

Iron and manganese affect plant photosynthesis, redox processes, nitrogen metabolism, and nucleic acid metabolism, i.e., in conifers. Excess manganese limits iron uptake and transport in plant tissues. This antagonism may also have the opposite effect, i.e., a high iron concentration reduces other metal uptake and activity [84,90]. In the tested samples, iron and manganese contents varied within wide ranges. The lowest contents of both metals were found in cones (No. 1–3) and pine wood (No. 4). In other samples, higher contents of iron and manganese were found (121–455 mg Fe/kg and 94–136 mg Mn/kg, respectively). Nkongolo et al. [89] found iron and manganese contents of 38–313 and 199–494 mg/kg in spruce branches, respectively. Kirchner et al. [87] noted Mn and Fe contents of 2.8–6.3 and 5.4–24.6 mg/kg in pine wood (*Pinus jeffreyi*), respectively. Galhetas et al. [55] found Mn and Fe contents of 330 mg Fe/kg and 32 mg Mn/kg respectively, in pine wood residues. Poikolainen [88] found pine bark Fe and Mn contents of 30–84 mg Fe/kg and 35–64 mg Mn/kg, respectively. For comparison, Liu et al. [6] found high Fe (141 mg/kg) and Mn (154 mg/kg) contents in pine bark. Aniszewska et al. [74] found a high iron content of 1130 mg/kg in pine and spruce seeds.

The presence of alkali metals in forestry residues is important in terms of thermal utility. It is known that there is a much higher content of alkali metals (mainly potassium and calcium) in biomass than in coal. When combined with sulphur and chlorine, alkali metals may cause high-temperature boiler corrosion. However, in the case of pyrolysis or gasification, alkali metals may be catalysts or catalytic precursors for these processes and may also affect the biomass in post-combustion flue gas self-desulphurisation [56]. 

The calcium content was low in cones (No. 1–3), with the lowest values of only 30 mg/kg found in spruce cones (No. 2). In other cone samples (No. 1 and 3), a higher Ca content of 200–300 mg/kg was found. In wood samples (No. 4–5), the calcium content was high (1200–1300 mg/kg). In samples of multispecies forest logging residues (No. 6–9), Ca content ranged from 2345 to 4149 mg/kg. In comparison, Galhetas et al. [55] found a calcium content of 3130 mg/kg in pine residues. Also, other authors have determined the following calcium contents in pine bark: 2319 [5] and 8292 mg/kg [10]. In spruce and pine needles, Smolander et al. [96] found a Ca content of 1640 and 3230 mg/kg, respectively. In pine seeds, Aniszewska et al. [74] noted a Ca content of 1167 mg/kg. A very high Ca content was determined by Cetin et al. [13] in spruce (*Picea pungens*), and depended on the anatomical parts of the plant tested: 7767–37,607 (unwashed needles), 12,272–26,236 (bark), and 4100–20,566 mg/kg (branches).

Magnesium is a basic component of chlorophyll. In conifers, it is also present in many enzymes [90]. 

Magnesium content in the tested samples was 253–537 mg/kg in cones (No. 1–3), 168–184 mg/kg in wood (No. 4–5), and 442–603 mg/kg in multispecies logging residues (No. 6–9), respectively. For comparison, Nkongolo et al. [89] found a magnesium content of 494–854 mg/kg in spruce branches. Galhetas et al. [55] determined a magnesium content of 400 mg/kg in pine wood residues. An Mg content of ca. 500 mg/kg was found in pine bark [5,10], and in the needles of spruce and pine, an Mg content of 1010 and 920 mg/kg respectively, was found [96]. In pine seeds, Aniszewska et al. [74] noted an Mg content of 3303 mg/kg. 

In biomass, with regard to alkali metals, potassium (K) content is usually the highest. Conifers use K in enzymatic processes and cell growth, in protein synthesis, photosynthesis, and for the correct initiation and termination of system functioning [90]. In the tested cones (No. 1–3), K content ranged from 2610 to 4154 mg/kg. In wood (No. 4–5), in contrast to other residues (No. 6–9), K content was found to be much lower (1073–1943 mg/kg). In comparison, Galhetas et al. [55] found a K content of 890 mg/kg in pine wood residues. For pine bark, Liu et al. [10] and Cutillas-Barreiro et al. [5] determined a K content of 1357 and 738 mg/kg, respectively. Smolander et al. [96] reported significant differences in the K content of pine and spruce needles, 4070 and 930 mg/kg, respectively. For pine seeds, Aniszewska et al. [74] determined the highest potassium content, among other alkali metals, of 6171 mg/kg.

Sodium is the last alkali metal to be considered in this paper. In the current study, sodium content was 57–160 mg/kg in cones (No. 1–3), 124–176 mg/kg in wood (No. 4–5), and 34–162 mg/kg in other residues (No. 6–9). In comparison, Galhetas et al. [55] found a sodium content of 240 mg/kg in pine wood residues, and Aniszewska et al. [74] found an Na content of 70 mg/kg in pine seeds. Other authors determined a sodium content of 20–94 [81] and 202 mg/kg in pine bark [10]. 

Table 6 presents the analysis of variance for the tested samples. ANOVA (Table 6) showed that the material type has a significant impact on the carbon content of the test samples (*p* < 0.05). Three homogeneous groups of average values were determined (HSD Tukey’s test, *p* > 0.05). According to the ANOVA (*p* = 0.91, Table 4), the average hydrogen content does not depend on material type. Statistically significant differences between the average content of sulphur and nitrogen occur in relation to the test material, as confirmed by ANOVA and the Tukey test (*p* < 0.05). ANOVA (*p* < 0.05) found a significant material type influence on the ash content. Average homogeneity tests confirmed that there is one large homogeneous group (*p* > 0.05), including cones and wood (No. 1–5) and one type of residues (No. 7), for which the differences in the average content are not statistically significant and additionally, there are two smaller groups. With regard to ash, ANOVA (*p* < 0.05) confirmed the influence of material type on the calorific value. Three homogenous groups (*p* > 0.05) with average values were determined, with small differences in values between them. With regard to all heavy metals, ANOVA (*p* < 0.05) revealed the significant influence of residue type on metal content. On the basis of post-hoc tests, homogeneous groups (*p* > 0.05) with average values marked with the letters a, b, c, etc., in Table 4 were separated. The content of alkali metals depends on the material type, as confirmed by ANOVA (*p* < 0.05), and post-hoc tests allowed for the determination of homogeneous groups (*p* > 0.05) with average values for individual materials. 

On the basis of the obtained results, it may be concluded that the elementary composition of forestry residues was similar to that obtained by other authors. All of the samples were characterised by a similar calorific value. Large differences were found in ash content in the tested samples. The lowest content of ash was found in cones and wood, while its highest content was found in mixed residue samples. The highest contents of heavy metals were found in round wood with bark (No. 5) and branch and tree top logging residues (No. 6–9). Higher contents of metals (Pb, Ni, Cr, Fe, and Mn) were found in the sample of round wood with bark (No. 5) vs. bark-free wood (No. 4). This may be due to the diversity of such biomass containing both wood and bark, leaves, needles, and various types of impurities, which may have been present during plant growth as well as harvesting, chipping, or storage. The lowest contents of heavy metals were found in cones (No. 1–3) and bark-free pine wood (No. 4), as confirmed by the literature data [61]. The differences in the composition of the tested forestry residues (No. 6–9) may have been influenced by tree age, the anatomical part being tested, and the natural condition of the environment. However, in the test samples, the content of heavy metals did not exceed the values provided by other authors, which might suggest that the tested waste was not contaminated. The content of alkali metals differed according to the type of forestry residue tested. For all of the samples, the highest alkali contents were found for potassium and calcium, which is consistent with the data provided by other authors. Alkali metal content is an important parameter from the point of view of their thermal use, as they may cause high-temperature boiler corrosion. 

The low content of heavy metals in the analysed waste suggests that they will not have a negative impact on their use as adsorbents or fuel. However, alkali metals such as Na and K in waste are considered only in terms of their use as fuel. A possible effect of cellulose and lignin treating with the use of hydroxides (NaOH, KOH) may be an increase in alkali content in forest waste. For this reason, the alkali content does not affect the adsorption process.

## 4. Conclusions

The elementary composition and calorific value of the forestry residues depend on the type and composition of the biomass, and the results did not differ significantly from the literature data. The waste was not contaminated with heavy metals (except Cr), therefore its application for energy purposes or adsorbent production should not have a negative impact on the environment.

After performing an analysis of the composition and content of the pollutants, it may be concluded that the tested forestry residues may be considered as potential substrates for the production of biomaterials or biofuel. Direct combustion, which should only be carried out in boilers designed for biomass combustion, is the exception to the above statement. However, the most appropriate direction of energy use of these wastes is temperature conversion, e.g., gasification or pyrolysis. Combustion may be carried out in specially designed biomass boilers, whereas ash with a high content of alkali metals may be used to neutralize acidic soil or wastewater. In order to reduce the ash content, untreated material, such as wood in pieces or as cones, should be used. Treatment and storage of logging residues may cause its mineral contamination, and consequently an increase in the ash content and a decrease in the calorific value.

Forest biomass residues are a suitable option for the development of biomaterials and biofuels, from the point of view of sustainable development. Unfortunately, their variable composition is a challenge for further development, especially for energy use. It should be noted that biomaterials or biofuels from forest waste are not of such high quality compared to wood. The advantage of forest waste is its low price and high availability. Research is still needed to increase their quality and reduce their heterogeneity, such as by segregating them at the source in the forest. An important aspect of using forest waste is its collection. In Poland, forest residue biomass is wasted to a significant degree due to inefficient use and unsustainable exploitation. Therefore, an important aspect of the use of forest waste is the high degree of its recovery in order to substitute wood.

## Figures and Tables

**Table 2:** Description of tested samples.

Sample	Type	Species Composition	Place of Processing
1	Cones	Pine	After the seed extraction
2	Cones	Spruce	After the seed extraction
3	Cones	Larch	After the seed extraction
4	Wood pieces without bark, sawmill residues	Pine	Fragmented in the recipient’s area
5	Round wood in bark, S4 grade	Scots pine	Fragmented in the recipient’s area
6	Logging residues, M2 grade	60% pine, 30% beech, 10% larch, admixture: ash, spruce, and hornbeam	Fragmented in the forest area
7	Logging residues, M2 grade, baling	50% pine, 50% spruce	Fragmented in the recipient’s area
8	Logging residues, M2 grade	70% spruce, 20% oak, 10% pine and linden	Fragmented in the forest area
9	Logging residues, M2 grade	50% spruce, 20% alder, 30% spruce brushwood	Fragmented in the forest area

Note: S4, M2—designation of wood grade in accordance with Regulation No. 51 of the Director General of the State Forests of 30 September 2019 on the technical conditions used in the trade of wood raw material in the State Forests National Forest Holding.

**Table 3:** The characteristics of tested samples (n = 3).

Sample	Elementar Analysis (% wt.)				Ash (% wt.)	Q_i_ (MJ/kg)
	C	H	N	S		
1	46.52 ^a,b^ ± 3.12	6.62 ^a^ ± 1.22	0.59 ^a^ ± 0.09	0.12 ^e^ ± 0.02	0.9 ^a^ ± 0.1	18.58 ^a,b^ ± 0.26
2	37.46 ^c^ ± 2.51	6.08 ^a^ ± 1.12	0.69 ^a,b^ ± 0.11	0.07 ^b^ ± 0.01	1.8 ^a,b^ ± 0.1	18.45 ^a^ ± 0.34
3	44.68 ^a,b,c^ ± 2.99	6.32 ^a^ ± 1.16	0.78 ^a,b^ ± 0.12	0.04 ^a,b^ ± 0.01	2.0 ^a,b^ ± 0.2	19.14 ^a,b,c^ ± 0.36
4	50.12 ^b^ ± 3.36	6.27 ^a^ ± 1.15	0.85 ^a,c^ ± 0.13	0.01 ^c,d^ ± 0.00	1.0 ^a^ ± 0.2	19.22 ^a,b,c^ ± 0.31
5	49.48 ^a,b^ ± 3.32	6.11 ^a^ ± 1.12	0.80 ^a,c^ ± 0.12	<0.005	1.3 ^a,b^ ± 0.2	19.35 ^b,c^ ± 0.40
6	43.02 ^a,b,c^ ± 2.88	5.53 ^a^ ± 1.02	1.32 ^b^ ± 0.20	0.05 ^a,b^ ± 0.01	5.3 ^c^ ± 0.2	18.91 ^a,b,c^ ± 0.48
7	47.37 ^a,b^ ± 3.17	6.13 ^a^ ± 1.13	0.94 ^a,b^ ± 0.15	0.03 ^a,d^ ± 0.01	2.1 ^a,b^ ± 0.2	19.54 ^c^ ± 0.23
8	41.10 ^a,c^ ± 2.75	5.37 ^a^ ± 0.99	0.97 ^a,b^ ± 0.15	0.05 ^a,b^ ± 0.01	4.6 ^c^ ± 1.7	18.62 ^a,b^ ± 0.58
9	44.49 ^a,b,c^ ± 2.98	5.70 ^a^ ± 1.05	1.30 ^b,c^ ± 0.20	0.03 ^a,c,d^ ± 0.00	2.8 ^b^ ± 0.2	19.05 ^a,b,c^ ± 0.29

Note: ^a, b, c^, the same letters indicate homogenous groups at *p* = 0.05, Q_i_ is calorific value.

**Table 4:** Content of heavy metals and nutrients (mg/kg DM) in the tested samples (n = 3).

Sample	Hg	Cd	Pb	Cu	Zn	Ni	Cr	Mo	Co	Fe	Mn	Ca	Mg	K	Na
1	0.010 ^e^ ± 0.000	<0.5 ^a^	1.5 ^a^ ± 0.0	2.3 ^b,c^ ± 0.0	12.1 ^a^ ± 0.2	1.4 ^b^ ± 0.0	2.8 ^a^ ± 0.0	11 ^a^ ± 0.2	<1.0 ^d^	67 ^a^ ± 1	36 ^d^ ± 0	185 ^a,b^ ± 3	285 ^b^ ± 3	4154 ^f^ ± 33	57 ^g^ ± 1
2	0.005 ^a^ ± 0.000	<0.5 ^a^	5.6 ^a^ ± 0.1	3.2 ^e^ ± 0.1	22.6 ^b^ ± 0.5	3.5 ^a^ ± 0.1	2.9 ^a^ ± 0.0	19 ^f^ ± 0.3	<1.0 ^d^	63 ^a^ ± 1	67 ^c^ ± 1	30 ^a^ ± 1	537 ^c^ ± 5	2747 ^c^ ± 22	137 ^c^ ± 1
3	0.006 ^a,b,c^ ± 0.000	<0.5 ^a^	10.5 ^a^ ± 0.1	2.4 ^c^ ± 0.0	14.4 ^a^ ± 0.3	1.8 ^b^ ± 0.0	2.8 ^a^ ± 0.0	14 ^a,b^ ± 0.2	<1.0 ^d^	32 ^c^ ± 0	51 ^b^ ± 1	274 ^b^ ± 3	253 ^b^ ± 3	2610 ^c^ ± 21	160 ^d^ ± 2
4	0.006 ^a,b,c^ ± 0.000	<0.5 ^a^	2.4 ^a^ ± 0.0	1.7 ^b^ ± 0.0	10.8 ^a^ ± 0.2	<1.0 ^e^	3.2 ^a^ ± 0.0	29 ^d,e^ ± 0.4	1.2 ^a^ ± 0.0	52 ^a,c^ ± 1	59 ^b,c^ ± 1	1241 ^c^ ± 16	184 ^a^ ± 2	533 ^a^ ± 4	124 ^b,c^ ± 1
5	0.006 ^b,c,d^ ± 0.000	<0.5 ^a^	150 ^c^ ± 1.5	4.4 ^a^ ± 0.1	11.7 ^a^ ± 0.2	9.3 ^d^ ± 0.2	31.4 ^e^ ± 0.3	22 ^g^ ± 0.3	<1.0 ^d^	138 ^b,d^ ± 2	109 ^f^ ± 1	1307 ^c^ ± 17	168 ^a^ ± 2	457 ^a^ ± 4	176 ^e^ ± 2
6	0.005 ^a,b^ ± 0.000	<0.5 ^a^	153 ^c^ ± 1.5	7.1 ^d^ ± 0.1	47.7 ^d^ ± 1.0	6.0 ^c^ ± 0.1	14.3 ^b^ ± 0.1	25 ^c^ ± 0.4	1.7 ^b^ ± 0.0	149 ^b^ ± 20	126 ^a^ ± 10	4107 ^d^ ± 53	603 ^d^ ± 6	1943 ^e^ ± 16	107 ^a^ ± 1
7	0.008 ^d^ ± 0.000	<0.5 ^a^	74.6 ^d^ ± 0.7	4.4 ^a^ ± 0.1	21.4 ^b^ ± 0.4	4.0 ^a^ ± 0.1	14.0 ^b^ ± 0.1	27 ^c,d^ ± 0.4	1.4 ^c^ ± 0.0	121 ^d^ ± 2	94 ^e^ ± 1	2345 ^e^ ± 30	442 ^e^ ± 4	1073 ^b^ ± 9	34 ^f^ ± 0
8	0.007 ^c,d^ ± 0.000	<0.5 ^a^	136 ^b^ ± 1	6.9 ^d^ ± 0.1	58.1 ^e^ ± 1.2	6.4 ^c^ ± 0.1	16.3 ^d^ ± 0.1	15 ^b^ ± 0	1.9 ^b^ ± 0.0	455 ^e^ ± 7	133 ^a^ ± 1	4149 ^d^ ± 54	581 ^d^ ± 6	1695 ^d^ ± 14	162 ^d,e^ ± 2
9	0.006 ^a,b,c,d^ ± 0.000	2.2 ^b^ ± 0.2	130 ^b^ ± 1	5.0 ^a^ ± 0.1	27.8 ^c^ ± 0.6	3.4 ^a^ ± 0.1	11.2 ^c^ ± 0.1	32 ^e^ ± 1	1.0 ^a^ ± 0.0	153 ^b^ ± 2	136 ^a^ ± 1	3206 ^f^ ± 42	500 ^c^ ± 5	1137 ^b^ ± 9	114 ^a,b^ ± 1

Note: ^a, b, c, d, e, f, g^—the same letters indicate homogenous groups at *p* = 0.05, DM—dry mass.

**Table 5:** Heavy metals limits in composts and biofuels (wood chips, pellets, briquettes).

Metal	Agricultural Use as First Class of Compost Quality *	Energy Use **	
		Briquettes and Pellets (Class A2)	Wood Chips (Class B1)
Cd (mg/kg DM)	5	0.5	2
Pb (mg/kg DM)	350	10	10
Cu (mg/kg DM)	300	10	10
Zn (mg/kg DM)	1500	100	100
Ni (mg/kg DM)	100	10	10
Cr (mg/kg DM)	300	10	10
Hg (mg/kg DM)	No limited	0.1	0.1

Note: *—first class quality of compost form municipal waste according to BN–89/9103–090 [91]; **—according to ISO 17225–2, 3, 4 [93,94,95]; A2 (bark, logging residue, chemical untreated wood residue); B1 (chemical untreated wood residue, forest plantation, and other virgin wood); DM—dry mass.

**Table 6:** Analysis of variance (ANOVA) results.

Parameter		Effect			Error		F	*p*-Value
	SS	df	MS	SS	df	MS		
C	391.5	8	0.2	0.4	18	0.0	9.0	0.00
H	3.0	8	0.5	22.1	18	1.2	0.4	0.91
N	1.5	8	0.2	0.4	18	0.0	9.0	0.00
S	0.03	8	0.0	0.0	18	0.0	34.3	0.00
Ash	62.4	8	7.8	6.6	18	0.4	21.3	0.00
Q_i_	5,577,632.6	8	697,204.1	5,054,363.2	36	140,399.0	5.0	0.00
Hg	0.0	8	0	0.00	18	0.00	33.4	0.00
Pb	114,720.3	8	14,340.0	373.9	18	20.8	690.4	0.00
Cu	93.5	8	11.6	0.7	18	0.04	299.0	0.00
Zn	6878.4	8	859.8	28.0	18	1.6	553.3	0.00
Ni	146.6	7	20.9	1.6	16	0.1	214.1	0.00
Cr	2173.1	8	271.6	3.5	18	0.2	1411.1	0.00
Mo	1270.2	8	158.8	17.3	18	1.0	165.2	0.00
Co	1.6	4	0.4	0.1	10	0.01	67.2	0.00
Fe	390,474.5	8	48,809.3	979.4	18	54.4	897.1	0.00
Mn	34,478.7	8	4309.8	409.2	18	22.7	189.6	0.00
Ca	65,083,065.1	8	8,135,383.1	88,370.3	18	4909.5	1657.1	0.00
Mg	719,048.8	8	89,881.1	3910.2	18	217.2	413.8	0.00
K	34,497,036.5	8	4,312,129.6	85,986.4	18	4777.0	902.7	0.00
Na	55,078.8	8	6884.8	450.8	18	25.0	274.9	0.00

Note: SS—sum of squares; df—degrees of freedom; MS—mean squares.

## Data Availability

Data sharing is not applicable to this article.
